# Case Report: Advanced Skeletal Muscle Imaging in S-Adenosylhomocysteine Hydrolase Deficiency and Further Insight Into Muscle Pathology

**DOI:** 10.3389/fped.2022.847445

**Published:** 2022-04-08

**Authors:** Danijela Petković Ramadža, Ivana Kuhtić, Kamelija Žarković, Hanns Lochmüller, Mislav Čavka, Ida Kovač, Ivo Barić, Maja Prutki

**Affiliations:** ^1^Department of Pediatrics, University Hospital Centre Zagreb, Zagreb, Croatia; ^2^School of Medicine, University of Zagreb, Zagreb, Croatia; ^3^Department of Radiology, University Hospital Centre Zagreb, Zagreb, Croatia; ^4^Department of Pathology, University Hospital Center Zagreb, Zagreb, Croatia; ^5^Children's Hospital of Eastern Ontario Research Institute, Ottawa, ON, Canada; ^6^Division of Neurology, Department of Medicine, The Ottawa Hospital, Brain and Mind Research Institute, University of Ottawa, Ottawa, ON, Canada; ^7^Department of Rehabilitation and Orthopaedic Devices, University Hospital Centre Zagreb, Zagreb, Croatia

**Keywords:** S-Adenosylhomocysteine hydrolase, S-Adenosylhomocysteine hydrolase deficiency, myopathy, magnetic resonance imaging with spectroscopy, skeletal muscle

## Abstract

**Introduction:**

S-Adenosylhomocysteine hydrolase deficiency (SAHHD) is a rare inherited multisystemic disease with muscle involvement as one of the most prominent and poorly understood features. To get better insight into muscle involvement, skeletal muscles were analyzed by magnetic resonance imaging (MRI) and MR spectroscopy (MRS) in three brothers with SAHHD in the different age group.

**Method:**

The study was based on analysis of MRI and MRS of skeletal muscles of the lower and the proximal muscle groups of the upper extremities in three SAHHD patients.

**Results:**

Three siblings presented in early infancy with similar signs and symptoms, including motor developmental delay. All manifested myopathy, more pronounced in the lower extremities and the proximal skeletal muscle groups, and permanently elevated creatine kinase. At the time of MRI and MRS study, the brothers were at the age of 13, 11, and 8 years, respectively. MRI revealed lipid infiltration, and the MRS curve showed an elevated muscle lipid fraction (higher peak of lipid), which increased with age, and was more prominent in the proximal skeletal muscles of the lower extremities. These results were consistent with muscle biopsy findings in two of them, while the third patient had no specific pathological changes in the examined muscle tissue.

**Conclusions:**

These findings demonstrate that an accessible and non-invasive method of MRI and MRS is useful for an insight into the extent of muscle involvement, monitoring disease progression, and response to treatment in SAHHD.

## Introduction

S-adenosylhomocysteine hydrolase (SAHH, EC 3.3.1.1) is a methionine cycle enzyme that catalyzes the hydrolysis of S-adenosylhomocysteine (AdoHcy) to adenosine and homocysteine (1). SAHH has a critical role in methylation processes and represents a key regulator of cellular transmethylation (2, 3). Pathogenesis of SAHH deficiency (SAHHD, OMIM 613752) is not fully understood, but it is assumed that elevation of AdoHcy and inhibition of methyltransferases play a significant role in causing biochemical and clinical abnormalities (4).

SAHHD has been reported in 15 patients (4–15). It is a multisystemic, clinically variable, autosomal recessive inherited metabolic disorder characterized by psychomotor delay, myopathy, and in various combinations liver dysfunction (4). Myopathy is one of the dominant features of SAHHD. Pathogenesis still needs to be elucidated, but there are several possible mechanisms (16, 17). There are only rare reports on muscle histology in SAHHD patients. Histology findings showed variability in fiber size, with necrotizing fibers and areas of regeneration (4, 5, 7).

MR spectroscopy (MRS) is a non-invasive method of metabolic imaging with magnetic resonance imaging (MRI), which detects a quantified signal of water, lipids, and other metabolites in the tissue, possibly representing the metabolism of interest. With water-suppression scheme, a fraction of lipids can be analyzed with more accuracy. In muscle, the intracellular and extracellular lipid fractions can be analyzed separately. Intramyocellular lipids (IMCL) are located near mitochondria, while extramyocellular lipids (EMCLs) are in adipocytes between muscle cells (18–20).

Molecular identification of specific metabolic markers is potentially useful for characterization of musculoskeletal abnormalities to help guide treatment decisions and follow-up (21–23). A few studies in the literature have explored the role of MRS lipid content in musculoskeletal imaging for muscle diseases (18, 19, 24, 25). Furthermore, lipid infiltration has been associated with disease progression, age, and clinical functional tests in Duchenne muscular dystrophy (DMD) (26). Using single-voxel ^1^H-MRS, a measure dependent on lipid infiltration, shows a higher value in DMD compared with controls, indicating increased muscle damage and inflammation/edema, which can be useful in monitoring disease progression (27–29).

In this case series, MRI and MRS findings of skeletal muscles in the lower and upper extremities of three siblings with SAHHD were analyzed. The results were partially presented as a poster at the Annual Symposium of the Society for the Study of Inborn Errors of Metabolism 2015 (30).

## Materials and Methods

All procedures performed in this case series involving human participants were in accordance with the ethical standards of the institutional and/or national research committee and with the 1964 Helsinki Declaration and its later amendments or comparable ethical standards.

The study was based on analysis of MRI and MRS of skeletal muscles of the lower and the proximal muscle groups of the upper extremities in three SAHHD patients. MRI and MRS were performed on the same day in three brothers at the age of 13, 11, and 8 years, respectively. MRI and MRS were acquired on a 3T scanner (Siemens Healthcare, Erlangen, Germany) using a four-element “body matrix” receiver coil and a circularly polarized (CP) body transmit coil.

On MRI, T2, T2 with fat suppression (slice: 4 mm, Dist: 5.2 mm, TR: 3,600 ms, TE: 92 ms) and T1 (slice: 5 mm, Dist: 6.5 mm, TR: 500 ms, TE: 15 ms) sequences were made in axial, coronary, and sagittal planes. Axial T2-weighted anatomic images (TR/TE 3,000 ms/30 ms, FOV 20 cm, slice thickness 7 mm, acquisition time 4 min) were collected to provide a guide for spectroscopy voxel localization within the vastus lateralis muscle, soleus muscle, and biceps brachii muscle. Prior to data collection, field homogeneity was optimized using linear, manual shimming. The voxel was positioned in the muscles with attention to avoid blood vessels, subcutaneous and other fat, and bone. For each voxel, a single-voxel Point-REsolved Spectroscopy Sequence (PRESS) [TR 2 s; TE 135 ms, voxel size 1 × 1 × 1 cm (1 cm^3^), 128 averages, acquisition time 4 min 20 s] spectrum was acquired with a four-pulse CHESS water-suppression scheme, followed by two acquisitions without water suppression (16 averages, scan time 40 s), one collected with “body matrix” receive and the other with the CP-transmit coil used as received.

Fatty infiltration of the lower extremities and the proximal muscle groups of the upper extremities was graded using semiquantitative and quantitative methods. The semiquantitative method using MR images (based on the morphological findings) entailed consensus scoring by two radiologists blinded to patient data to minimize bias, resulting in the agreement of both readers. Semiquantitative method was performed at the largest cross-sectional area of each muscle; we used the scale described by Kim et al. (31), as follows: grade 0, homogeneous muscle signal intensity without fatty infiltration; grade 1 (minimal), predominantly homogeneous muscle signal intensity with minimal scattered fatty infiltration (often seen in soleus muscle); grade 2 (mild), mild fatty infiltration with additional patchy areas of intramuscular high T1 signal intensity involving <30% of muscle bulk; grade 3 (moderate), moderate fatty infiltration involving 30–60% of muscle bulk, and preserved differentiation between muscle and subcutaneous fat; and grade 4 (severe), severe fatty infiltration involving more than 60% of muscle bulk with loss of demarcation between muscle and subcutaneous fat.

Quantitative measures of muscle fatty infiltration were obtained by determining the amount of intramuscular adipose tissue on MRS in the vastus lateralis muscle, soleus muscle, and biceps brachii muscle. The results were compared between the brothers.

## Results

### Case Description

The patients are children of healthy, unrelated parents, born at term after a normal pregnancy. The definite diagnosis of SAHHD was made by sequence analysis of the *AHCY* gene revealing two pathogenic mutations: the maternally derived c.336G>A (p.Trp112Ter) and the paternally derived c.428A>G (p.Tyr143Cys).

All three brothers had similar symptoms: psychomotor delay, myopathy, mild hepatopathy, disturbed coagulation, behavioral problems, and cognitive impairment. Myopathy was the most prominent symptom unamenable to dietary treatment, which resulted in significant decrease in S-adenosylmethionine (AdoMet) and AdoHcy, while CK and aminotransferases were permanently elevated (≥10 × of the upper limit of normal). At the time of skeletal muscle MRI and MRS, all patients had hypotonia, muscle weakness (more prominent in the proximal muscles, especially in the lower extremities), fatigability, and obesity due to low physical activity. Nevertheless, they all walked unassisted, and were able to perform everyday chores.

#### Patient 1

The index patient manifested psychomotor delay since birth. He presented at the age of 8 months with severely delayed development, hypotonia, more in the lower than in the upper extremities, convergent strabismus, and microcephaly. Diagnostic workup showed elevated CK and aminotransferases, low albumin, and prolonged prothrombin time (the latter as signs of impaired liver synthetic function), and myopathic potentials on electromyography. Skeletal muscle biopsy was performed at the age of 13 months. Histology revealed variability in fiber size with few necrotizing fibers undergoing phagocytosis and some basophilic regenerating fibers. Histochemistry demonstrated no pathological changes. Electron microscopy (EM) showed numerous myelin figures of different sizes and shapes in muscle fibers and extracellularly, and numerous enlarged and abnormally shaped mitochondria within some fibers. Immunofluorescence muscle staining did not point to the etiopathogenesis of the disease (see Supplementary Material 1). Brain MRI revealed white matter atrophy and impaired myelination. The diagnosis of SAHHD was confirmed at the age of 12.8 months by measuring low SAHH activity in red blood cells, fibroblasts, and liver, and confirming biallelic pathogenic mutations in the *AHCY* gene. Methionine-restricted diet and supplementation of phosphatidylcholine and creatine were started at the age of 13 months. The treatment resulted in marked decrease in AdoMet, AdoHcy, and methionine, and constant gradual clinical improvement. Patient became more alert and communicative, and muscle strength improved (4). He started to walk unassisted at age 19 months.

Second muscle biopsy was performed at 12.5 years. Specimen showed fiber variability, endomysial edema, and some fatty infiltration and inflammation between muscle fibers. Histochemistry demonstrated no significant changes. EM revealed membranous myelin-like figures (see detailed description and EM figures in Supplementary Material 2).

At the time of skeletal muscle MRI and MRS, the patient was 13 years old. He complained of fatigability and occasional lower leg muscle aches. For the clinical status, the patient had myopathic face, generalized muscle weakness, and hypotonia, predominantly in the lower limbs, hyporeflexia, waddling gait, and was unable to heel walk and squat. Muscle strength [Medical Research Council (MRC) scale] of the lower limbs was pelvic girdle (2 to −3/5), quadriceps muscle (−5/5), foot dorsiflexors (3/5), and plantar flexors (4/5). CK fluctuated between 7,486 and 10,970 U/L (reference range 75–280).

#### Patient 2

The second brother of the index patient manifested hypotonia since birth. At first clinical evaluation at 15 days of life, he had reduced spontaneous movements, generalized hypotonia, absent tendon reflexes, and elevated CK and aminotransferases. Brain MRI showed delayed myelination and frontotemporal atrophy. In contrast to his brother, this patient had neither manifesting liver disease nor coagulation disturbance. The diagnosis of SAHHD was confirmed at the age of 3.4 months by measuring elevated AdoMet and AdoHcy, low residual SAHH activity in red blood cells, and confirming biallelic family mutations in the *ACHY* gene. At the time of diagnosis, the boy had hypotonia, convergent strabismus, and developmental delay, although less severe than his older brother at corresponding age. Upon the diagnosis, treatment with low methionine diet and supplementation of phosphatidylcholine and creatine were started. It resulted in improving muscle strength, alertness, and spontaneous movements. In the subsequent period, strabismus disappeared, tendon reflexes (although week) could be elicited, and muscle hypotonia was less evident. Patient was able to sit unsupported at 10 months, and to stand and walk with support at 13 months. Control brain MRI performed 7 months after the treatment initiation showed almost normal myelination for age. The biopsy of the right deltoid muscle was performed at age 13.5 months, and histological examination revealed fairly normal muscle fibers except for slightly increased variation in fiber size. Immunohistochemistry was normal, and EM revealed a small number of myelin figures of different sizes and shapes, and focal myofibrillar degeneration in the subsarcolemmal regions (5). On long-term follow-up, this sibling manifested the best outcome considering muscle strength, endurance, and cognitive abilities.

At the time of MRI and MRS, the patient was 11 years old. He complained of fatigability and had muscle weakness, predominantly of the lower limbs, hypotonia and hyporeflexia, waddling gait, and positive Gowers sign. Muscle strength of the lower limbs (MRC scale) was pelvic girdle (3/5), quadriceps muscle (+4/5), foot dorsiflexors (+3/5), and plantar flexors (4/5). CK fluctuated between 5,419 and 26,369 U/L (reference range 75–280).

#### Patient 3

The third patient harbored the same pathogenic mutations of the *AHCY* gene as his brothers. He exhibited clear signs of myopathy already at birth: sluggishness, shallow breathing, floppiness, diminished spontaneous movements, and absent tendon reflexes. Comparison with his brothers regarding the presentation of their inherited disease was somewhat complicated, as he experienced mild perinatal hypoxia (Apgar scores 8 and 9), which might had contributed to the clinical symptoms. The patient had biochemical hallmarks of SAHHD: elevated AdoMet, AdoHcy, and CK. Low methionine diet and oral phosphatidylcholine supplementation were started at age 18 days. Creatine was added a month later. On the treatment, the patient gradually gained strength, became more alert, and had better social contact and spontaneous activity (6). Developmental milestones were delayed. He started to walk with support at 19 months of age.

A muscle tissue was taken during orthopedic procedure of the hip at the age of 5.4 years, and histopathology, immunohistochemistry (staining for spectrin, dystrophin, emerin, merosin, dysferlin, α-sarcoglycan, and α-actinin), and EM showed normal finding.

At the time of MRI and MRS, the patient was 8 years old. On clinical examination, he had positive Mingazzini and Gowers tests and waddling gate. The muscle strength of lower limbs (MRC scale) was pelvic girdle (−3/5), quadriceps muscle (+4/5), foot dorsiflexors (+3/5), and plantar flexors (4/5). CK fluctuated between 6,853 and 38,392 U/L (reference range 75–280).

### Magnetic Resonance Imaging and MR Spectroscopy Findings

**Figure 1:**
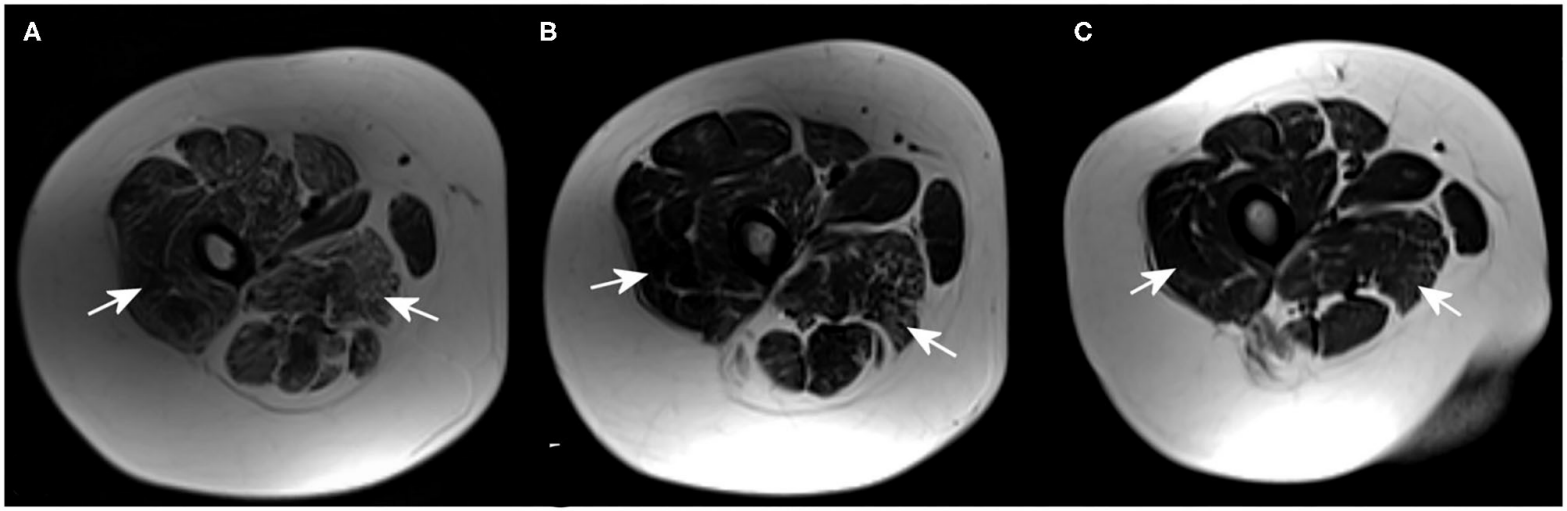
Magnetic resonance imaging (MRI) (axial T2-weighted images) of proximal muscles groups of the lower extremities showing the dominant fatty infiltration in the most affected muscles [vastus lateralis muscle and adductor magnus muscle (arrows)] in Patient 1 **(A)**, Patient 2 **(B)**, and Patient 3 **(C)**.

MRI revealed that the most affected were the posterior groups of proximal muscles of the lower extremities, followed by the distal muscles of the lower extremities, and the proximal muscles of the upper extremities. MRI showed an abnormal fatty infiltration in the proximal muscles of the lower extremities, especially in the posterior muscle group (vastus lateralis and adductor magnus). On the other hand, the gracilis, adductor longus, and brevis muscles were spared. The most pronounced changes were found in the oldest brother (Patient 1), and the least in the youngest brother (Patient 3) (Figure 1 and Table 1).

**Table 1:** Fatty infiltration of the muscles on magnetic resonance imaging (MRI) using the semiquantitative method (29).

	**Skeletal muscle**		
	**Proximal muscle groups of lower extremities**	**Distal muscle groups of lower extremities**	**Proximal muscle groups of upper extremities**
Patient 1	4	4	2
Patient 2	3	1	0
Patient 3	2	3	0

**Figure 2:**
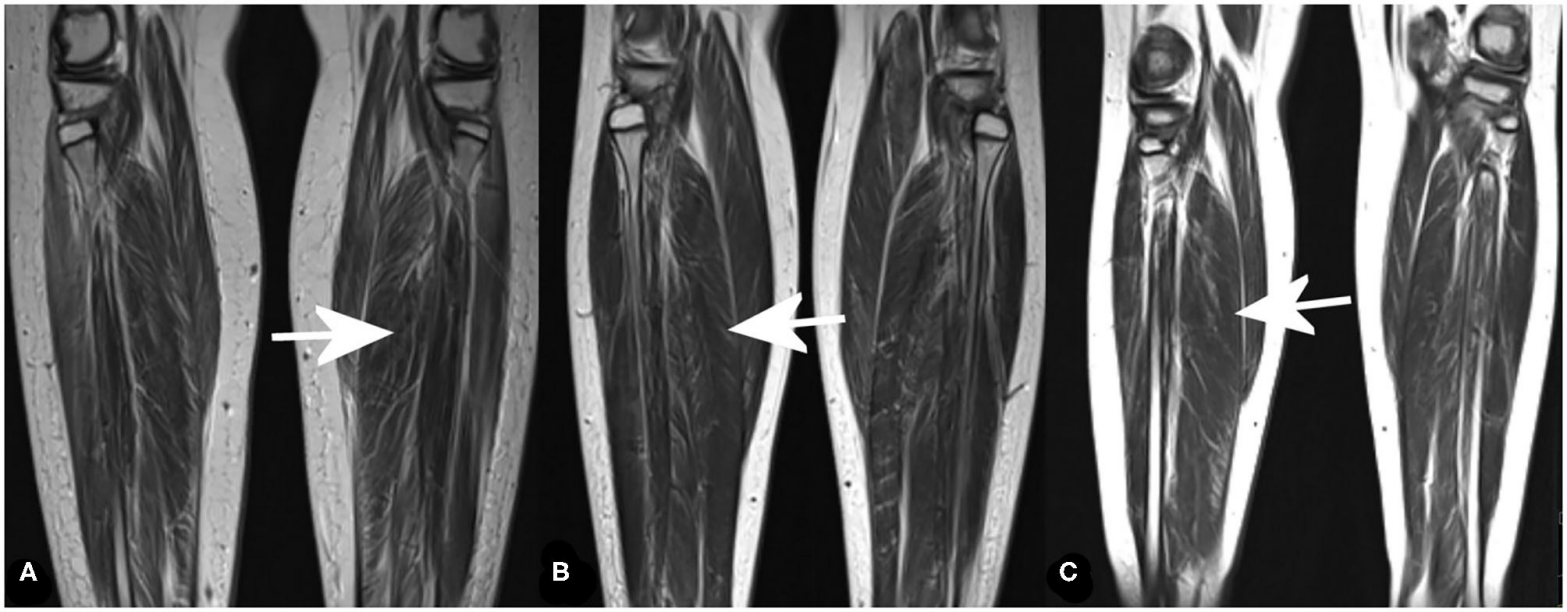
MRI of distal muscles groups of the lower extremities (coronal T2-weighted images) showing fatty infiltration in the most affected muscle [soleus muscle (arrows)] in Patient 1 **(A)**, Patient 2 **(B)**, and Patient 3 **(C)**.

In the distal muscles of the lower extremities, the most pronounced pathology was observed in the soleus and peroneus muscle. Again, the most prominent changes were found in the oldest brother (Patient 1), the minor changes were present in the middle brother (Patient 2), and in the youngest brother, and changes were moderate (Figure 2 and Table 1). There was significant edema in the soleus muscle without signs of pseudohypertrophy (muscle diameter was in the referral interval for age).

In the area of the proximal part of the upper extremities, MRI showed fatty infiltration of the biceps brachii muscle in the oldest brother (Patient 1). In the two younger brothers, the finding was normal (Table 1).

MRS with voxel placed within the vastus lateralis muscle of the proximal part of the lower extremity showed a high peak of EMCL (CH_2_ and CH_3_) (Table 2). The highest peak of EMCL was detected in the oldest brother (Patient 1), slightly lower in the youngest brother (Patient 3), and the lowest in the middle brother (Patient 2). No increase in IMCL (CH_2_ and CH_3_) was detected in either sibling.

**Table 2:** Lipid amplitude in MR spectroscopy (MRS) in vastus lateralis muscle and soleus muscle.

	**Skeletal muscle**					
**Ppm**	**Vastus lateralis muscle**			**Soleus muscle**		
	**Patient 1**	**Patient 2**	**Patient 3**	**Patient 1**	**Patient 2**	**Patient 3**
EMCL (CH_3_)—lipids	**67.5**	11.8	28.3	0	0	26.7
1.5 EMCL (CH_2_)—lipids	**250.5**	**54.7**	**132.8**	**191.9**	**43.5**	**50.7**
2.2 Methylene group of ester binding	**70**	11.5	23	**43.2**	9l.9	12.2

*EMCL, extramyocellular lipids*.

*The values higher than reference values are in bold*.

The elevated peak of EMCL (CH_2_) was detected in the soleus muscle in the distal part of the lower extremity of the oldest brother (Patient 1), slightly lower in the youngest brother (Patient 3), and again the smallest peak in the middle brother (Patient 2). EMCL (CH_3_) was detected only in the youngest sibling (Patient 3). No increase in IMCL (CH_2_ and CH_3_) was detected in either sibling.

MRS within the biceps brachii muscle in the proximal part of the upper extremity showed a slightly elevated peak of EMCL (CH_2_) in the oldest brother. The findings of the MRS in the two younger brothers were normal.

## Discussion

SAHHD is a rare multisystemic disease caused by disorder of the methionine cycle. Clinical presentation is variable, but myopathy is a constant feature (2, 16). It is still unclear how SAHHD affects muscles.

One of the proposed pathological mechanisms was choline depletion due to inhibition of methyltransferase responsible for conversion of phosphatidylethanolamine to phosphatidylcholine, a choline precursor (16). Choline availability affects muscle membrane lipid composition and intracellular lipid metabolism, and choline depletion causes accumulation of lipid droplets in muscle cells (32). We found no intracellular lipid accumulation in analyzed muscle tissue in any of our patients. MRS results are in accordance with that finding, showing normal IMCL in all three patients. Additionally, all our patients had persistently elevated CK despite phosphatidylcholine supplementation. All presented findings suggest that choline deficiency is not the leading cause of myopathy in SAHHD.

Although dietary treatment had no effect on serum CK, and did not reverse muscle disease, clinical evolution of myopathy in our patients may suggest at least partial improvement after initiation of methionine-restricted diet. Besides, some other SAHHD features are amenable to the dietary treatment and subsequent decrease in AdoHcy (e.g., white matter myelination) (4, 5). An important prerequisite for better outcome may be early recognition.

Corresponding to the muscle histology findings, Patient 1 had prominent changes on muscle MRI and MRS. On the other hand, muscle MRI and MRS revealed pathological changes, consistent with clinical and biochemical signs of myopathy, and also in the youngest sibling who had a normal muscle histology (with the remark that muscle biopsy was performed ~2.5 years before MRI and MRS analysis). Possible explanation for negative histology finding, besides different time period, is that the sample had not been taken from the clinically most affected muscles (muscle biopsy was obtained during orthopedic hip surgery). MRI and MRS may be more accurate in detecting muscle involvement in SAHHD than histology, as this method provides possibility to analyze all skeletal muscle groups of upper and lower extremities, and to detect changes despite the uneven distribution of muscle pathology. It is worth to stress that coagulopathy is an important feature, which may cause bleeding disorder in SAHHD; therefore, MRI and MRS may be an excellent substitute for an invasive muscle biopsy to evaluate muscle involvement. Also, the two diagnostic methods may be used complementarily, and imaging finding can be a guide in the decision on which muscle to biopsy.

Skeletal muscle MRI showed abnormal fatty infiltration, edema, and atrophy, predominantly in the proximal muscles of the lower extremities, in all three patients. The changes were most extensive in Patient 1 who also had the most severe myopathy among the three siblings. These findings were consistent with previously described skeletal muscle changes characteristic of muscular dystrophies, such as DMD (26, 27, 31, 33–37).

The MRS is a relatively easily available and non-invasive method, which measures the content of metabolites and lipids in observed skeletal muscles and demonstrates fatty infiltration. It showed that the content of lipids in target muscles was the most pronounced in the eldest brother. This may reflect the later diagnosis and treatment of the index patient or the progressive natural course of the disease.

In Patient 2, MRS showed a slightly lower proportion of lipids in muscles among the three siblings, but also more severe muscle edema. Clinically, Patient 2 had milder myopathy, attained developmental milestones earlier, and had less muscle histology changes than Patient 1. In addition, he manifested neither liver disease nor clotting disturbance at the time of diagnosis. Besides better muscle strength, he displayed the best cognitive outcome during a long-term follow-up. Although these lines of evidence may suggest that Patient 2 had milder phenotype, this conclusion is equivocal as he was diagnosed and treated earlier than his older brother. Patient 1 had been exposed to higher amounts of methionine and consequently higher amounts of AdoHcy during the first year of life, which probably led to significantly impaired transmethylation reactions in the developing body, and thus in the muscles. On the other hand, the fact that does not support this hypothesis is that the youngest brother, in whom treatment was started even earlier (in the newborn period), had more severe myopathy than Patient 2. MRI morphological findings of targeted muscles were almost equal in Patients 2 and 3, with more pronounced fatty infiltration of the distal lower extremities in Patient 3.

Overall, the most pronounced fatty infiltration was in the posterior muscle groups of the proximal lower extremities, followed by groups of the distal lower extremities, and only in the oldest brother in the proximal muscle groups of the upper extremities. These results show that fatty infiltration progresses from proximal toward the distal parts, primarily in the lower extremities, and correlates with clinical severity of myopathy.

## Conclusion

In conclusion, this is the first report of skeletal muscle fatty infiltration in SAHHD, which is a common finding in various other genetic muscle diseases, suggesting progressive loss of muscle mass due to impaired regeneration process (38). To the best of our knowledge, this is the first study describing comparative MRI and MRS findings in patients with myopathy due to SAHHD. Results presented in this study open the possibility of (1) gaining insight into the extent of muscle pathology in SAHHD, (2) monitoring the course and natural progression of the disease, and (3) evaluating the response to currently available treatment and possible future therapies with an accessible and non-invasive methods of MRI and MRS.

## Data Availability Statement

The raw data supporting the conclusions of this article will be made available by the authors, without undue reservation.

## Ethics Statement

The studies involving human participants were reviewed and approved by Clinical Hospital Center Zagreb. Written informed consent to participate in this study was provided by the participants' legal guardian/next of kin.

## Author Contributions

All authors listed have made a substantial, direct, and intellectual contribution to the work and approved it for publication.

## Funding

This work was partly funded by the grant Inherited Metabolic Diseases in Children of the University of Zagreb for 2014.

## Conflict of Interest

The authors declare that the research was conducted in the absence of any commercial or financial relationships that could be construed as a potential conflict of interest.

## Publisher's Note

All claims expressed in this article are solely those of the authors and do not necessarily represent those of their affiliated organizations, or those of the publisher, the editors and the reviewers. Any product that may be evaluated in this article, or claim that may be made by its manufacturer, is not guaranteed or endorsed by the publisher.

## Supplementary Material

Supplementary Table 1 Immunoflourescence staining on skeletal muscle of Patient 1.

Supplementary Table 2 Pathohistology of skeletal muscle tissue from Patient 1.
