# Supplementary material for: Case Report: Advanced Skeletal Muscle Imaging in S-Adenosylhomocysteine Hydrolase Deficiency and Further Insight Into Muscle Pathology
Source: Front Pediatr. 2022 Apr 8;10:847445. doi: 10.3389/fped.2022.847445 (PMC9026168; doi:10.3389/fped.2022.847445)
Supplement: Supplementary Table 1 — Immunoflourescence staining on skeletal muscle of Patient 1. [file Data_Sheet_1.docx]

**Immunofluorescence staining on skeletal muscle of Patient 1** **(muscle biopsy performed at the age of 13 months)**: normal expression of dystrophin (dys-1,2,3), alpha-sarcoglycan, beta-sarcoglycan, gamma-sarcoglycan, delta-sarcoglycan, alpha-dystroglycan, caveolin 3, collagen XI alpha-1, dysferlin, emerin, lamin A/C and merosin. Novocastra antibodies were used according to the supplier’s instructions. (Analysis was performed subsequently to the first publication of index patient - previously unpublished data)
