# Supplementary material for: Case Report: Advanced Skeletal Muscle Imaging in S-Adenosylhomocysteine Hydrolase Deficiency and Further Insight Into Muscle Pathology
Source: Front Pediatr. 2022 Apr 8;10:847445. doi: 10.3389/fped.2022.847445 (PMC9026168; doi:10.3389/fped.2022.847445)
Supplement: Supplementary Table 2 — Pathohistology of skeletal muscle tissue from Patient 1. [file Data_Sheet_2.pdf]

Pathohistology of skeletal muscle tissue from Patient 1 (muscle biopsy performed at the age of 12.5 years): Specimen showed slight variation in fiber size. Most nuclei were located under the sarcolemma and only sporadically demonstrated internal nuclei. In a longitudinally section some small fibers, scattered through the biopsy, showed chains of subsarcolemmal nuclei. Adipose tissue separated muscle fibers only in one small spot. Endomysial connective tissue network appeared delicate and had one small inflammatory infiltrate composed of CD68 and CD45 positive mononuclear cells around regenerating basophilic fibers with vesicular nuclei. Segmental, small subsarcolemmal fiber necrosis was visible only under ultrastructural analysis. There was a diffuse distribution of type1 and 2 fibers with slight predominance of type 1 fibers. Gomori trichrome, periodic acid-Schiff (PAS) and oil red showed regular staining. Reduced nicotinamide adenine dinucleotide dehydrogenase-tetrazolium reductase (NADH-TR), combined cytochrome oxidase and succinic dehydrogenase (SDH) and menadione- linked α-glycerophosphate dehydrogenase staining did not show abnormalities of the oxidative enzymes. Ultrastructural analysis showed mitochondrial swelling or vacuolization of cristae (Figure 1), collection of membranous myelin-like figures predominantly in subsarcolemmal region (Figure 2), and small areas of myofibrillar disorganization predominantly in subsarcolemmal region around nuclei. Sarcolemma of some atrophic fibers was folded in the extracellular space.

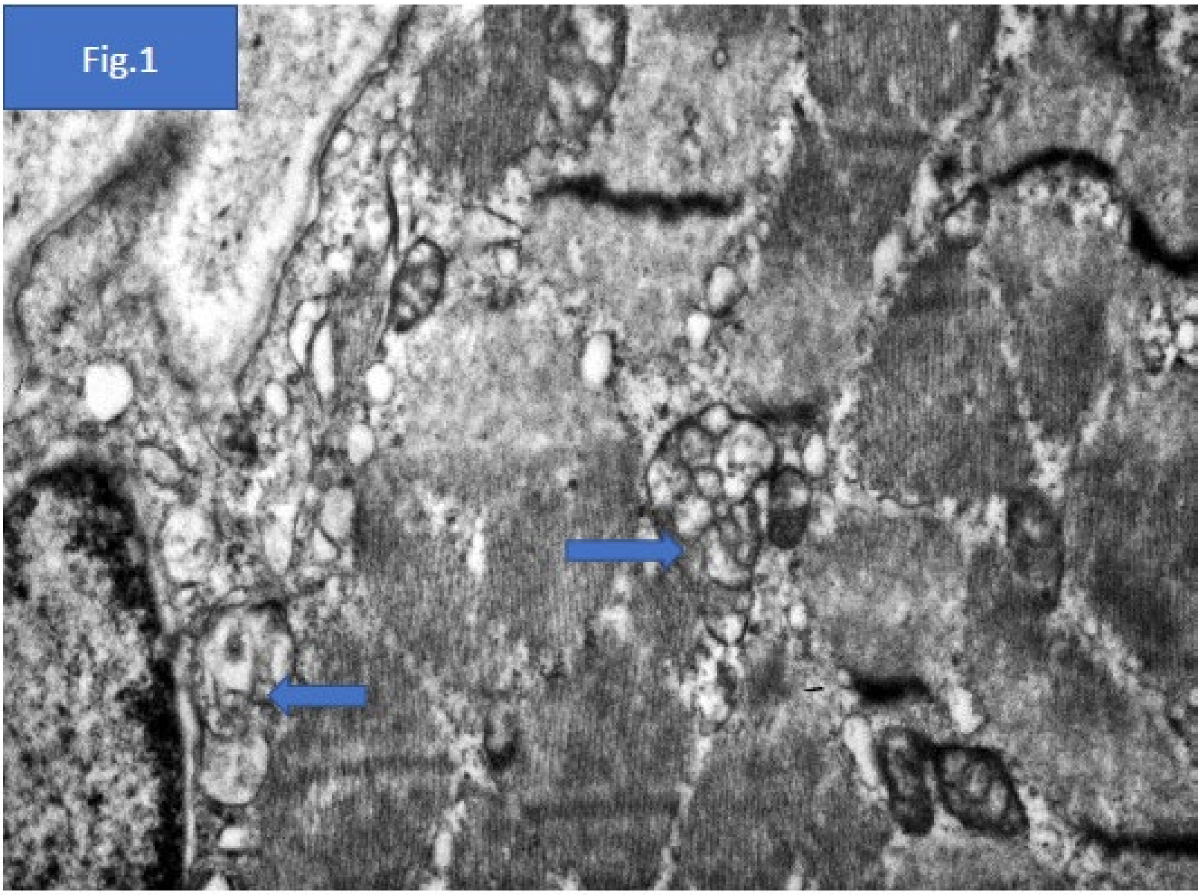

Figure 1. Electron photomicrograph showing mitochondrial swelling and vacuolization (arrows).

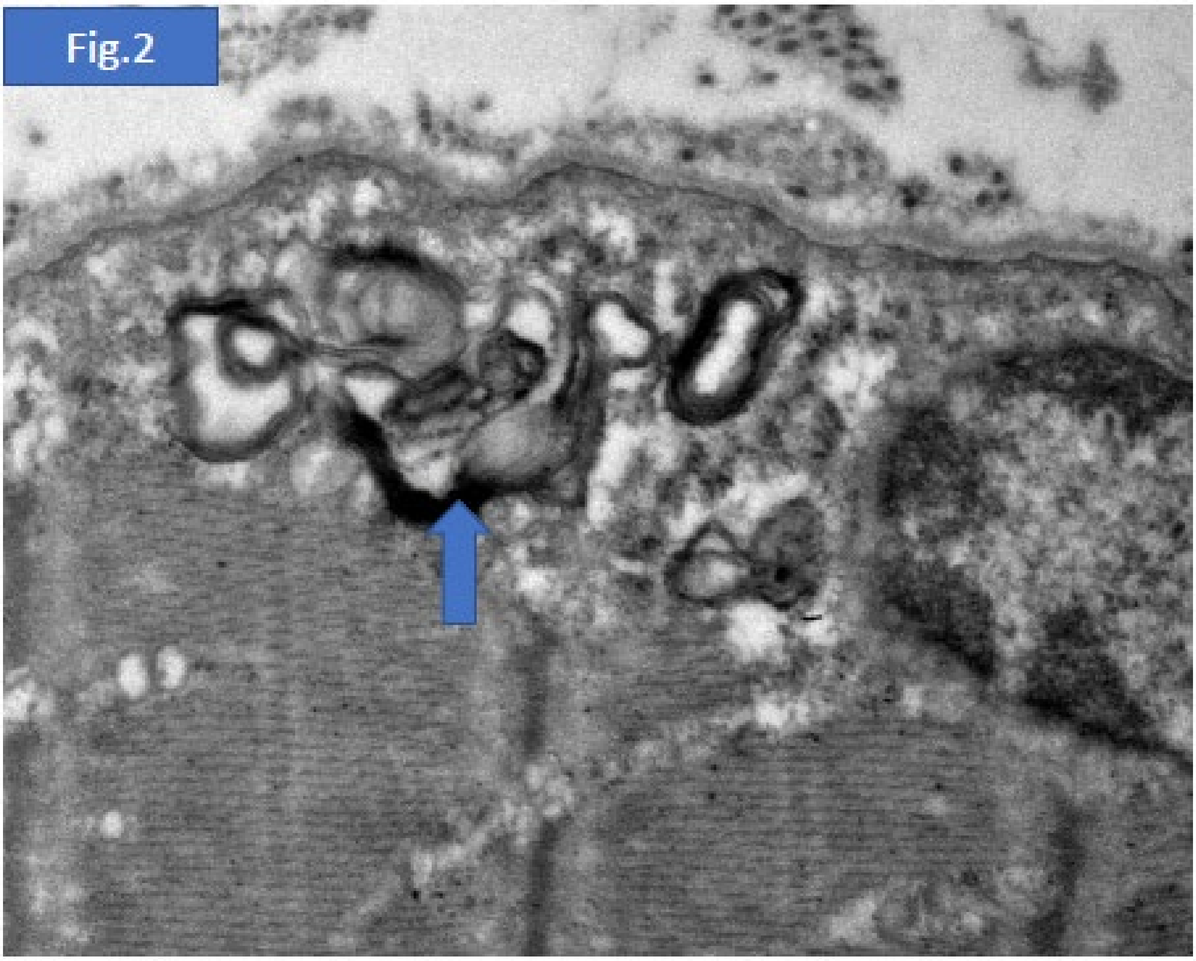

Figure 2. Electron photomicrograph showing myelin-like appearance in subsarcolemmal region (arrow).
